# Supplementary material for: Upregulation of ATP-purinergic P2×2 receptors in the cochlea over-amplifies hearing sensitivity leading to hyperacusis and attenuation by antagonists
Source: bioRxiv. 2026 Jun 22:2026.06.17.733049. Preprint. [Version 1] doi: 10.64898/2026.06.17.733049 (PMC13320770; doi:10.64898/2026.06.17.733049)
Supplement: Supplement 1 [file NIHPP2026.06.17.733049v1-supplement-1.pdf]

bioRxiv preprint doi: https://doi.org/10.64898/2026.06.17.733049; this version posted June 22, 2026. The copyright holder for this preprint (which was not certified by peer review) is the author/funder, who has granted bioRxiv a license to display the preprint in perpetuity. It is made available under a CC-BY 4.0 International license.

Supplementary figure legends:

Fig-S1. No apparent changes of P2x2 expressions in the auditory centers. **a**: A diagram of the afferent auditory system and locations of the auditory cortex (AC), inferior colliculus (IC), and cochlear nucleus (CN) in the mouse brain (the cerebellum was removed to visualize the CN). **b-d**: Volcano plots of gene upregulation and downregulation in the AC, IC, and CN in $Cx26^{+/-}$ mice in Bulk Poly(A) RNA-Seq. There were no significant changes of P2x2 expressions in these auditory centers in $Cx26^{+/-}$ mice.

Fig-S2. No apparent expression or upregulation of P2x2 expression in the auditory centers in WT and $Cx26^{+/-}$ mice examined by dPCR. The P2x2 expression was calculated from reference to the reference gene *Tfrc*. **a-b**: P2x2 expressions in AC, IC, CN, and cochlea in $Cx26^{+/-}$ mice. **c-d**: P2x2 expressions in the AC, IC, CN, and cochlea in WT mice. Panel **a&c** are the represented droplet plots of dPCR for P2x2 and the reference gene *Tfrc* in WT and $Cx26^{+/-}$ mice. There are no P2x2 expressions in the AC, IC, and CN in both $Cx26^{+/-}$ mice and WT mice. In the cochlea, P2x2 expressions referring to Tfrc are 0.28±0.02 and 0.12±0.01 in $Cx26^{+/-}$ mice and WT mice, respectively. In comparison with WT mice, the P2x2 expression in the $Cx26^{+/-}$ mouse cochlea has a significant increase ($P>0.001$, 2-tail t test).

Fig-S3. No changes of P2x7 expression in the auditory system in $Cx26^{+/-}$ mice. The expressions of P2x7 in the AC, IC, CN, and cochlea were examined by dPCR. **a**: Represented dPCR droplet plots of P2x7 expressions in the AC, IC, CN, and cochlea in $Cx26^{+/-}$ mice and WT mice. **b**: Fold changes of P2x7 expression in $Cx26^{+/-}$ mice.

bioRxiv preprint doi: https://doi.org/10.64898/2026.06.17.733049; this version posted June 22, 2026. The copyright holder for this preprint (which was not certified by peer review) is the author/funder, who has granted bioRxiv a license to display the preprint in perpetuity. It is made available under a CC-BY 4.0 International license.

Referring to WT mice, there are no significant changes of P2x7 expression in $Cx26^{+/-}$ mice (P>0.05, one-way ANOVA).

Fig. S4. No changes of Cx26 and P2x7 expressions in the cochlea after injection of P2x2 vectors. The expressions of Cx26, P2x2, and P2x7 in the right ears with P2x2 vector injection were measured by dPCR and normalized to those in the left ears without injection. P2x2 expression has a significant increase of 2.85±0.43 (n=10) referring to the referring to the left ears (P<0.01 2-tail t test), while the expressions of Cx26 and P2x7 are 0.94±0.17 and 0.96±0.15, respectively (P=0.75 and 0.81, respectively, 2-tail t test) and have no significant changes.

Fig. S5. No significant difference in ABR thresholds between $Cx26^{+/-}$ mice and WT mice after administration of PPADS. ABR was measured at 8 h after administration of PPADS. There were no significant differences in ABR thresholds between $Cx26^{+/-}$ mice and WT mice (P = 0.52 – 0.97, 2-tail t test).

Fig. S6. No significant effects on hearing function in the left control ear without P2x2 vector injection after administration of PPADS. Hearing functions in the left control ears were measured before and after 8 h of administration of PPADS by close-field recording. **a-c**: There were no significant changes in ABR, CM, and DPOAE in the left-ears without injection after administration of PPADS.

bioRxiv preprint doi: https://doi.org/10.64898/2026.06.17.733049; this version posted June 22, 2026. The copyright holder for this preprint (which was not certified by peer review) is the author/funder, who has granted bioRxiv a license to display the preprint in perpetuity. It is made available under a CC-BY 4.0 International license.

Fig. S7. PPADS has no significant effects on hearing function in the right ear with empty vector injection. Right ears were injected with AAV empty vectors without P2x2. ABR thresholds were measured with the close-field recording before and after 8 h of administration of PPADS.

Fig. S8. No significant differences of ABR thresholds between mice with P2x2 and empty vector injections after administration of PPADS. The P2x2 or empty vector was injected into only the right ear. ABR thresholds in the right ear were recorded before and after 8 h of administration of PPADS by close-field recording. **a**: ABR thresholds in the right ear with P2x2 or empty vector injection. In comparison with empty vector injections, the injection of P2x2 vectors significantly reduces ABR thresholds. *: $P<0.05$, **: $P<0.01$, 2-tail t test. **b**: There are no significant differences in ABR thresholds between P2x2 and empty vector injection ears after 8 h of administration of PPADS.

Fig-S9. No significant changes in prestin expression in Cx26 hetero-deletion mice and P2x2 vector injection mice. Prestin expression in the cochlea was examined by dPCR and normalized to the reference gene *Tfrc*. **a**: There are no significant changes of prestin in Cx26 hetero-deletion mice. **b**: There are no significant changes of prestin after injection of P2x2 vectors.

Fig-S10. P2x2 receptors are required for ATP modification on OHC electromotility. **a**: Immunofluorescent staining for P2x2 and Prestin in OHCs. Scale bar: 10 µm. **b-c**: ATP-evoked inward current in OHCs and absence in P2x2 KO mice. The OHC was held at -80

bioRxiv preprint doi: https://doi.org/10.64898/2026.06.17.733049; this version posted June 22, 2026. The copyright holder for this preprint (which was not certified by peer review) is the author/funder, who has granted bioRxiv a license to display the preprint in perpetuity. It is made available under a CC-BY 4.0 International license.

mV under the whole-cell recording configuration in patch clamp recording. Application of 50 μM ATP evoked an apparent inward current in OHC in WT mice but not in P2x2 KO mice. There was also no apparent current visible in I-V curve for voltage-step stimulation (panel **c**). **d-h**: There is no apparent effect of ATP on OHC electromotility in P2x2 KO mice. NLCs have no significant changes after perfusion of 50 μM ATP in a P2x2 KO mouse OHC. In comparison with control before perfusion, Q_{max}, z, V_{pk}, and NLC have no significant changes after perfusion of 50 μM ATP ($P>0.05$, 2-tail t test).

Fig-S11. No significant changes of ASR and OHC electromotility in P2x2 deficient mice. **a-c**: No significant changes of ASR in P2x2 KO mice. **d:** NLCs recorded from WT, $P2x2^{+/-}$ hetero-deletion, and $P2x2^{-/-}$ KO mice. Smooth lines represent fitting the first derivative of the Boltzmann equation. The fitting parameters are: Q_{max}=0.85 and 0.86 pC; z=0.85and 0.83; V_{pk}=-78.0 and -74.6 mV; C_{lin}=5.47 and 5.53 pF, respectively, for NLC recorded from WT, $P2x2^{+/-}$, and P2x2 KO mice. **e-h**: Parameters of NLC fitting in the WT, $P2x2^{+/-}$, and P2x2 KO mice. There are no significant changes in OHC electromotility in $P2x2^{+/-}$ and P2x2 KO mice with one-way ANOVA analysis.

bioRxiv preprint doi: https://doi.org/10.64898/2026.06.17.733049; this version posted June 22, 2026. The copyright holder for this preprint (which was not certified by peer review) is the author/funder, who has granted bioRxiv a license to display the preprint in perpetuity. It is made available under a CC-BY 4.0 International license.

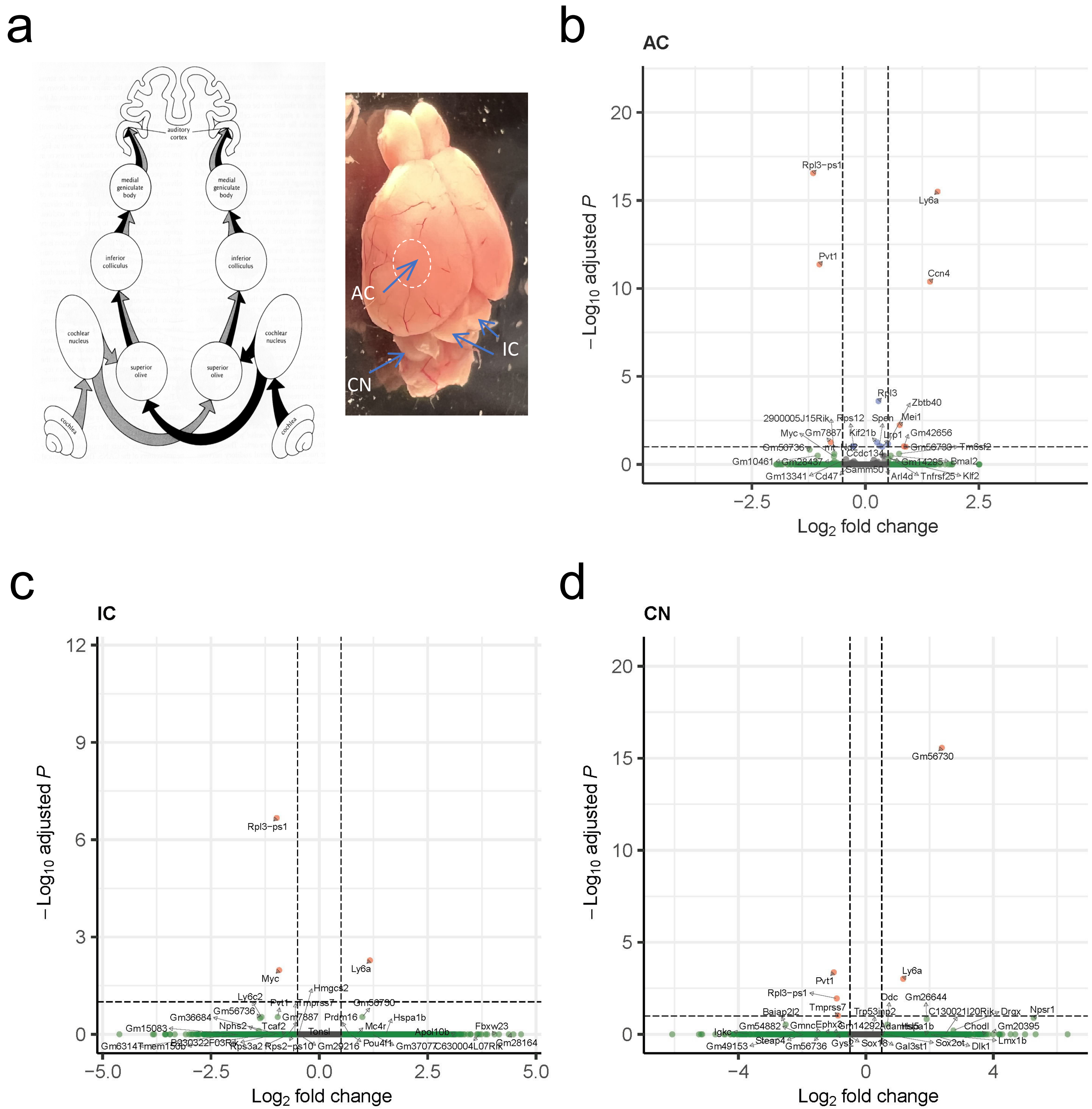

Fig. S1. No apparent changes of P2x2 expressions in the auditory center

bioRxiv preprint doi: https://doi.org/10.64898/2026.06.17.733049; this version posted June 22, 2026. The copyright holder for this preprint (which was not certified by peer review) is the author/funder, who has granted bioRxiv a license to display the preprint in perpetuity. It is made available under a CC-BY 4.0 International license.

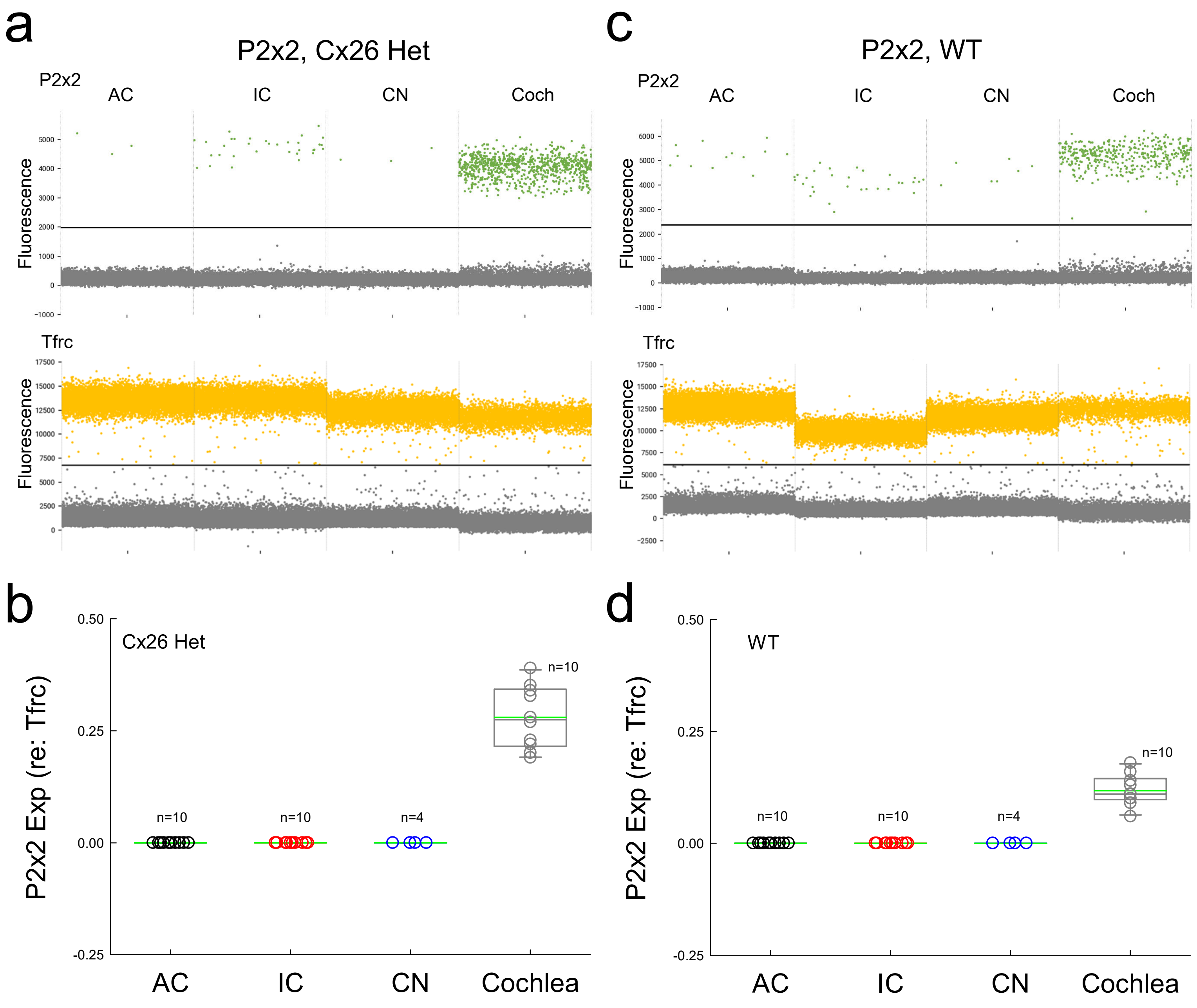

Fig. S2. No apparent expression and upregulation of P2x2 expression in the auditory center in WT and $Cx26^{+/-}$ mice

bioRxiv preprint doi: https://doi.org/10.64898/2026.06.17.733049; this version posted June 22, 2026. The copyright holder for this preprint (which was not certified by peer review) is the author/funder, who has granted bioRxiv a license to display the preprint in perpetuity. It is made available under a CC-BY 4.0 International license.

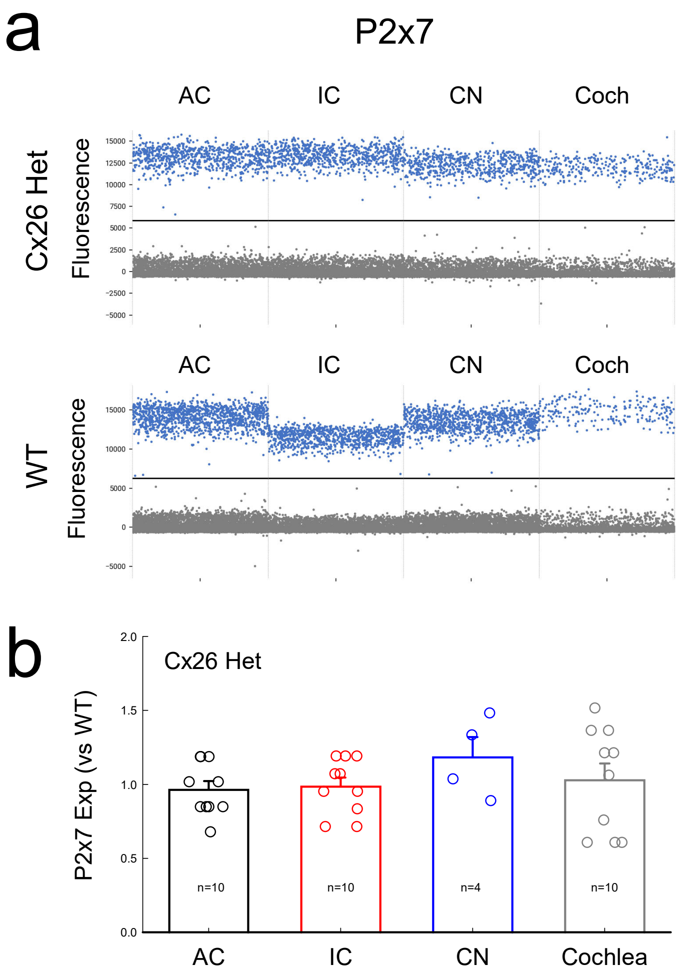

Fig. S3. No changes of P2x7 expression in the auditory system in Cx26 Het mice

bioRxiv preprint doi: https://doi.org/10.64898/2026.06.17.733049; this version posted June 22, 2026. The copyright holder for this preprint (which was not certified by peer review) is the author/funder, who has granted bioRxiv a license to display the preprint in perpetuity. It is made available under a CC-BY 4.0 International license.

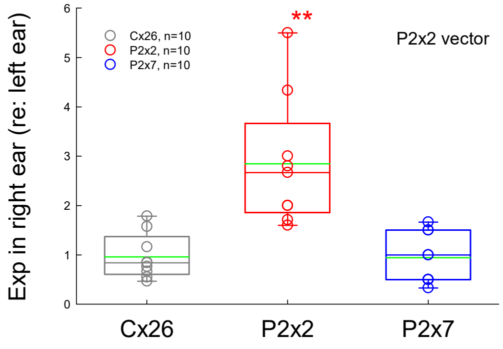

Fig. S4. No changes of Cx26 and P2x7 expressions after injection of P2x2 vectors

bioRxiv preprint doi: https://doi.org/10.64898/2026.06.17.733049; this version posted June 22, 2026. The copyright holder for this preprint (which was not certified by peer review) is the author/funder, who has granted bioRxiv a license to display the preprint in perpetuity. It is made available under a CC-BY 4.0 International license.

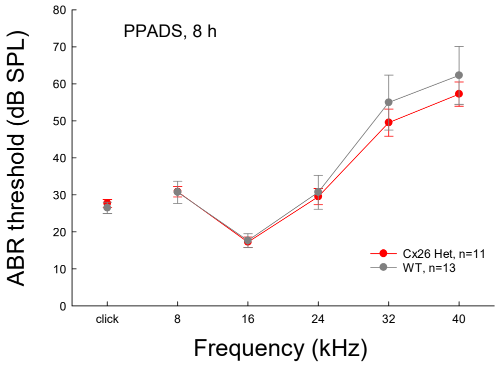

Fig. S5. No significant difference in ABR thresholds between Cx26 Het mice and WT mice after administration of PPADS

bioRxiv preprint doi: https://doi.org/10.64898/2026.06.17.733049; this version posted June 22, 2026. The copyright holder for this preprint (which was not certified by peer review) is the author/funder, who has granted bioRxiv a license to display the preprint in perpetuity. It is made available under a CC-BY 4.0 International license.

PPADS effects on the left ear without vector injection

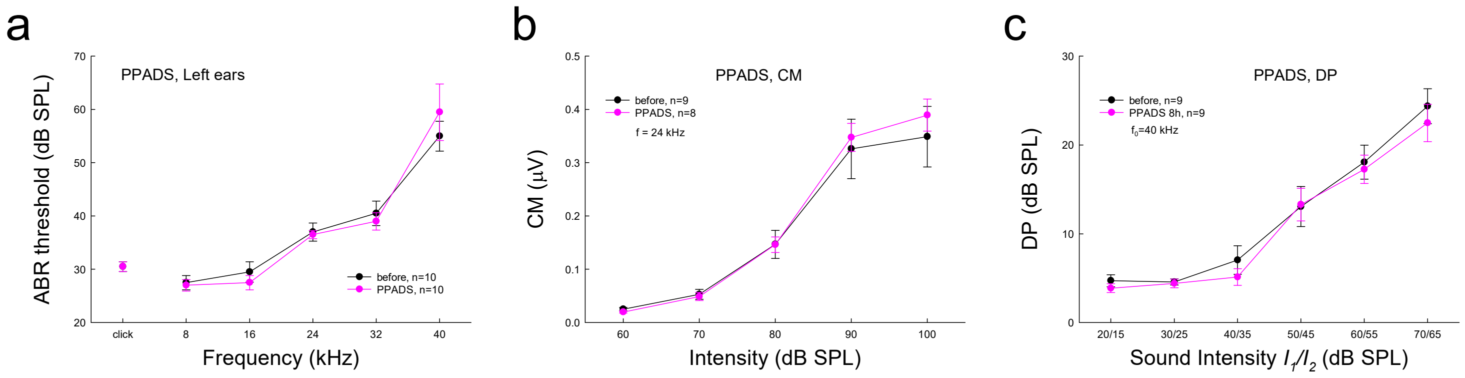

Fig S6. No changes of hearing function in the left-ear without P2x2 vector injection after administration of PPADS

bioRxiv preprint doi: https://doi.org/10.64898/2026.06.17.733049; this version posted June 22, 2026. The copyright holder for this preprint (which was not certified by peer review) is the author/funder, who has granted bioRxiv a license to display the preprint in perpetuity. It is made available under a CC-BY 4.0 International license.

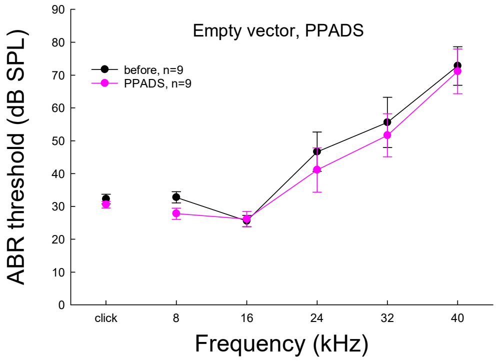

Fig S7. No changes of hearing function after administration of PPADS in the right-ear with empty vector injection

bioRxiv preprint doi: https://doi.org/10.64898/2026.06.17.733049; this version posted June 22, 2026. The copyright holder for this preprint (which was not certified by peer review) is the author/funder, who has granted bioRxiv a license to display the preprint in perpetuity. It is made available under a CC-BY 4.0 International license.

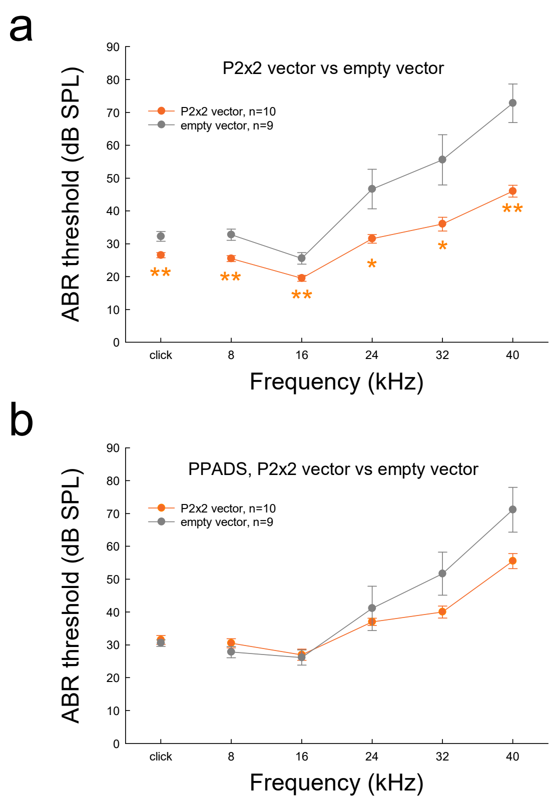

Fig S8. Comparison of ABR thresholds between P2x2 and empty vector injection mice in pre- and post-injection of PPADS

bioRxiv preprint doi: https://doi.org/10.64898/2026.06.17.733049; this version posted June 22, 2026. The copyright holder for this preprint (which was not certified by peer review) is the author/funder, who has granted bioRxiv a license to display the preprint in perpetuity. It is made available under a CC-BY 4.0 International license.

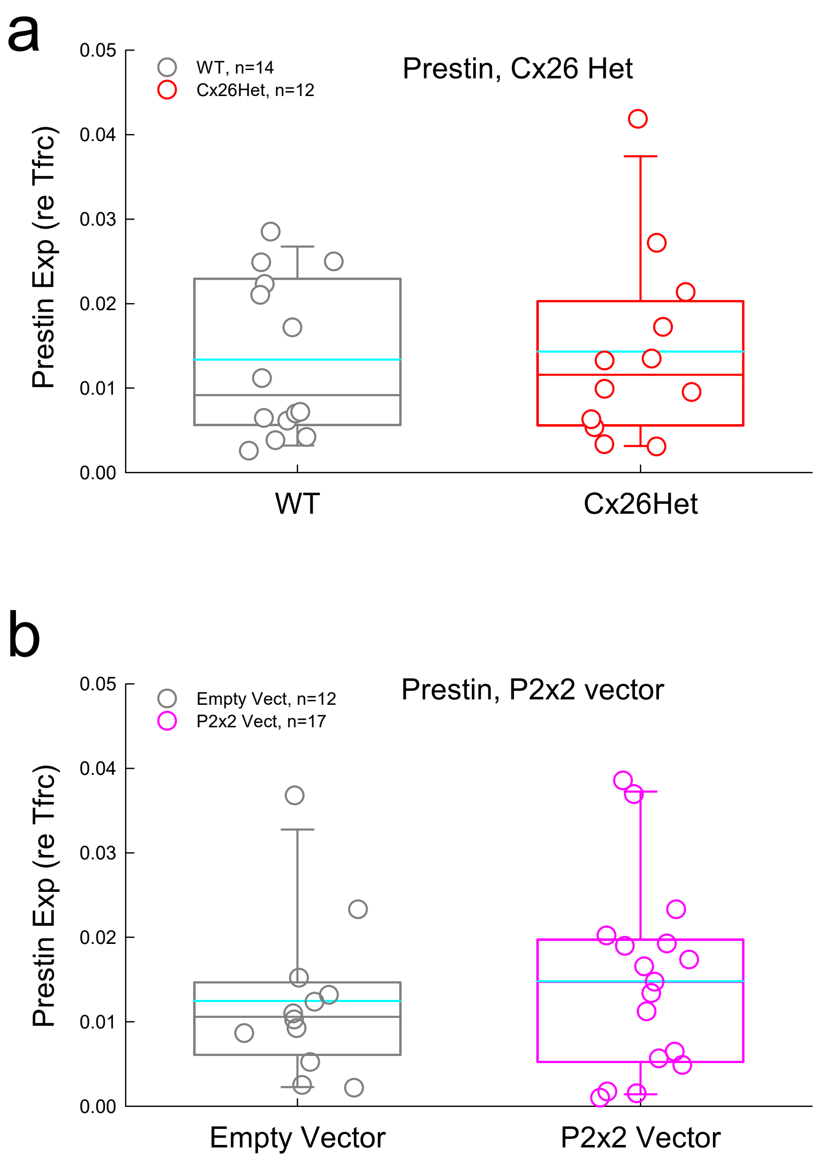

Fig. S9. No changes in prestin expression in Cx26 hetero-deletion mice and P2x2 vector injection mice

bioRxiv preprint doi: https://doi.org/10.64898/2026.06.17.733049; this version posted June 22, 2026. The copyright holder for this preprint (which was not certified by peer review) is the author/funder, who has granted bioRxiv a license to display the preprint in perpetuity. It is made available under a CC-BY 4.0 International license.

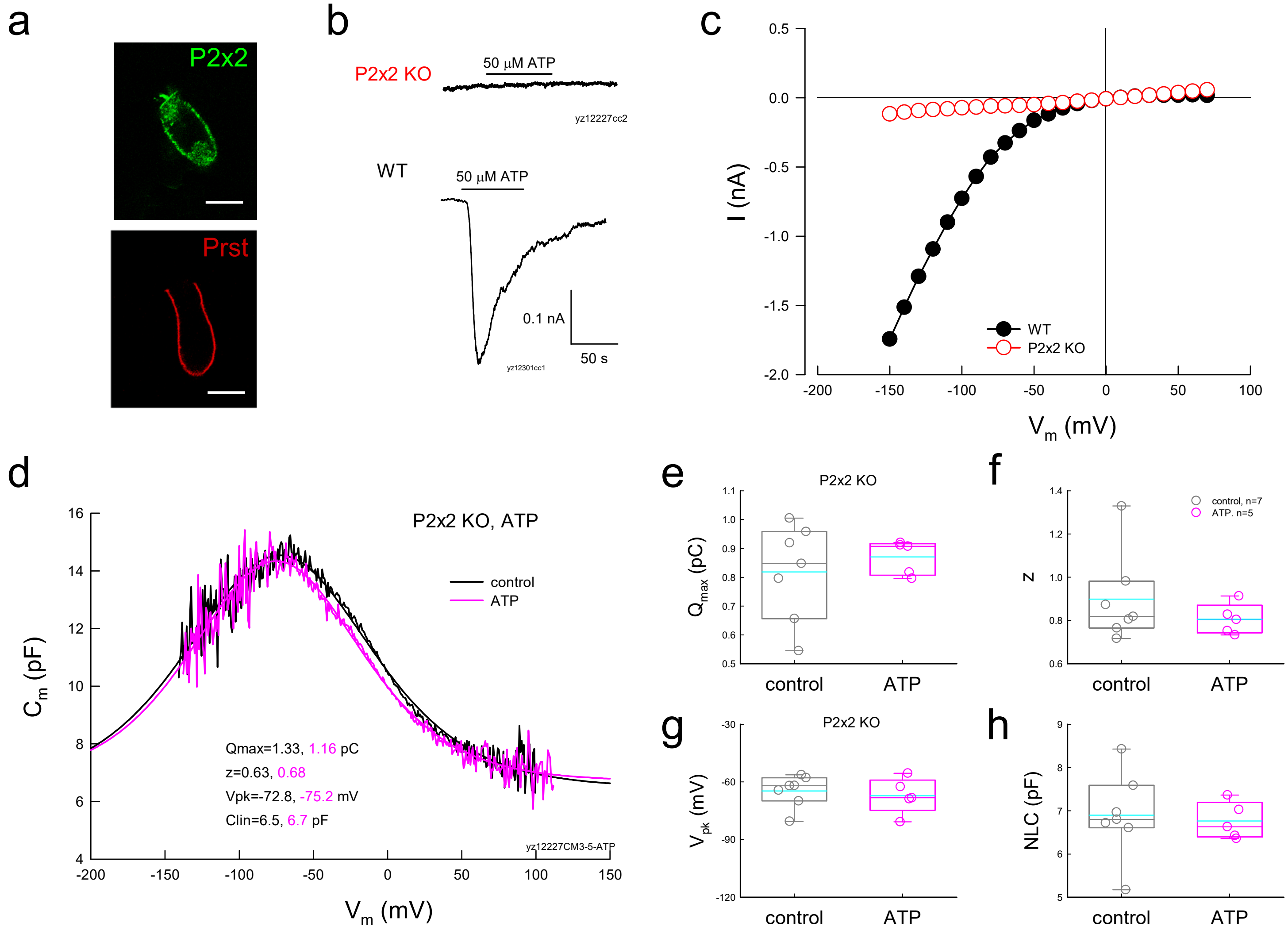

Fig. S10. P2X2 is required for modification of ATP on OHC electromotility

bioRxiv preprint doi: https://doi.org/10.64898/2026.06.17.733049; this version posted June 22, 2026. The copyright holder for this preprint (which was not certified by peer review) is the author/funder, who has granted bioRxiv a license to display the preprint in perpetuity. It is made available under a CC-BY 4.0 International license.

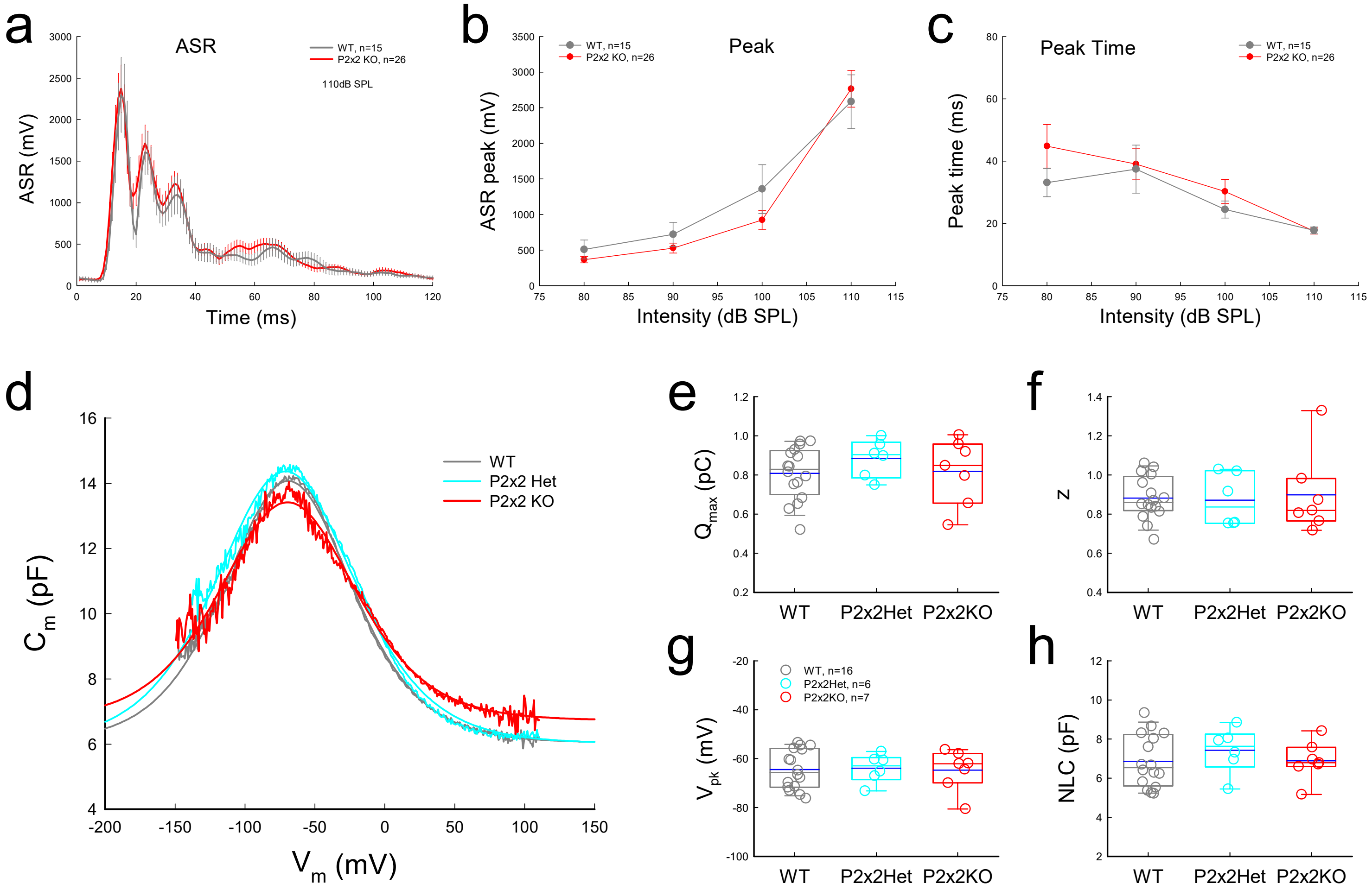

Fig. S11. No significant changes of ASR and OHC electromotility in P2x2 deficient mice
